# Whole-Genome Sequences of *Streptococcus thermophilus* Strains TH1435 and TH1436, Isolated from Raw Goat Milk

**DOI:** 10.1128/genomeA.01129-13

**Published:** 2014-01-16

**Authors:** Laura Treu, Veronica Vendramin, Barbara Bovo, Stefano Campanaro, Viviana Corich, Alessio Giacomini

**Affiliations:** Department of Agronomy, Food, Natural Resources, Animals and Environment (DAFNAE), University of Padua, Padua, Italya; Department of Biology, University of Padua, Padua, Italyb

## Abstract

We report the genome sequences of two *Streptococcus thermophilus* strains, TH1435 and TH1436, isolated from raw goat milk devoted to the production of artisanal cheese in the Friuli-Venezia Giulia region in Italy. The genome sequences of these two quickly acidifying strains are the first available genome sequences of *S. thermophilus* strains isolated in Italy.

## GENOME ANNOUNCEMENT

*Streptococcus thermophilus* is a homofermentative thermophilic lactic acid bacterium (LAB) used worldwide as a starter culture for the manufacture of a variety of fermented dairy products (1). To date, the complete genomes at the chromosome level of *S. thermophilus* strains CNRZ1066 (2), JIM8232 (3), LMD-9 (4), LMG18311 (2), MN-ZLW-002 (5), and ND03 (6) have been published. However, *S. thermophilus* TH1435 and TH1436 represent the first cases of *S. thermophilus* strains isolated in Italy and the only ones from goat milk.

*S. thermophilus* TH1435 and TH1436, collected from two alpine huts (malghe) from raw goat milk used for the artisanal production of Italian cheese, are capable of rapidly lowering the pH of milk, which represents a key technological feature for the dairy industry. Moreover, strain TH1436 is capable of utilizing galactose, while TH1435 lacks this feature.

Whole-genome sequencing of strains TH1435 and TH1436 was performed with an Illumina MiSeq sequencer at the Ramaciotti Centre, Sydney, Australia. Genomic libraries were prepared using the Nextera XT kit Illumina (Illumina, Inc., San Diego, CA), which produced a mean insert size between 800 and 1,200 bp. A total of 776,373 and 1,527,857 paired-end reads (2 × 250 bp) were generated and gave 134- and 159-fold coverages of the TH1435 and TH1436 genomes, respectively. Approximately 85% of these reads were assembled into 36 and 28 large scaffolds, respectively, using a manually curated consensus of assemblies obtained using version 1.2.10 of the Velvet software (7) and version 2.8 of the 454 Newbler Assembler (454 Life Sciences, Branford, CT). The draft genome of *S. thermophilus* TH1435 is a single circular chromosome of 1,750,348 bases in length, with a mean G+C content of 38.9% and two putative plasmids individuated by BLAST match (scaffolds 33 and 35). The draft genome of strain TH1436 is a single circular chromosome of 1,780,473 bases, with a mean G+C content of 39.0%; no plasmid sequences were detected for this strain.

Genome annotation was performed using RAST (8) and by the NCBI Prokaryotic Genomes Annotation Pipeline (9). A total of 1,925 coding sequences (CDSs) and 47 structural RNAs were predicted in strain TH1435, while 1,899 CDSs and 48 RNAs were found in strain TH1436. Additionally, several phage-associated sequences were identified, 12 genes related to transposases and 7 clusters of regularly interspaced short palindromic repeats (CRISPRs) in both strains.

A comparison against the complete genome of *S. thermophilus* CNRZ1066 revealed that TH1435 and TH1436 contain 38 common predicted CDSs that are absent in the reference strain. Furthermore, 31 CDSs specific to TH1435 and 18 specific to TH1436 are present. The sequences of particular interest include a unique gene cluster for the biosynthesis of bacteriocins, resistance to antibiotics, and toxic compounds, as well as several gene clusters that indicate that TH1435 harbors at least one prophage.

### Nucleotide sequence accession numbers.

The data from these whole-genome shotgun projects were deposited in GenBank under the accession no. AYSG00000000 for *S. thermophilus* TH1435 and AYTT00000000 for *S. thermophilus* TH1436. The versions described in this paper are versions AYSG01000000 and AYTT01000000, respectively.

## References

[1] Iyer R Tomar S Uma Maheswari T Singh R 2010 *Streptococcus thermophilus* strains: Multifunctional lactic acid bacteria. Int. Dairy J. 20:133–141. 10.1016/j.idairyj.2009.10.005

[2] Bolotin A Quinquis B Renault P Sorokin A Ehrlich SD Kulakauskas S Lapidus A Goltsman E Mazur M Pusch GD Fonstein M Overbeek R Kyprides N Purnelle B Prozzi D Ngui K Masuy D Hancy F Burteau S Boutry M Delcour J Goffeau A Hols P 2004 Complete sequence and comparative genome analysis of the dairy bacterium *Streptococcus thermophilus*. Nat. Biotechnol. 22:1554–1558. 10.1038/nbt1034 15543133 PMC7416660

[3] Delorme C Bartholini C Luraschi M Pons N Loux V Almeida M Guédon E Gibrat JF Renault P 2011 Complete genome sequence of the pigmented *Streptococcus thermophilus* strain JIM8232. J. Bacteriol. 193:5581–5582. 10.1128/JB.05404-11 21914889 PMC3187418

[4] Makarova K Slesarev A Wolf Y Sorokin A Mirkin B Koonin E Pavlov A Pavlova N Karamychev V Polouchine N Shakhova V Grigoriev I Lou Y Rohksar D Lucas S Huang K Goodstein DM Hawkins T Plengvidhya V Welker D Hughes J Goh Y Benson A Baldwin K Lee JH Díaz-Muñiz I Dosti B Smeianov V Wechter W Barabote R Lorca G Altermann E Barrangou R Ganesan B Xie Y Rawsthorne H Tamir D Parker C Breidt F Broadbent J Hutkins R O’Sullivan D Steele J Unlu G Saier M Klaenhammer T Richardson P Kozyavkin S Weimer B Mills D 2006 Comparative genomics of the lactic acid bacteria. Proc. Natl. Acad. Sci. U. S. A. 103:15611–15616. 10.1073/pnas.0607117103 17030793 PMC1622870

[5] Kang X Ling N Sun G Zhou Q Zhang L Sheng Q 2012 Complete genome sequence of *Streptococcus thermophilus* strain MN-ZLW-002. J. Bacteriol. 194:4428–4429. 10.1128/JB.00740-12 22843572 PMC3416250

[6] Sun Z Chen X Wang J Zhao W Shao Y Wu L Zhou Z Sun T Wang L Meng H Zhang H Chen W 2011 Complete genome sequence of *Streptococcus thermophilus* strain ND03. J. Bacteriol. 193:793–794. 10.1128/JB.01374-10 21131489 PMC3021231

[7] Zerbino DR Birney E 2008 Velvet: algorithms for *de novo* short read assembly using de Bruijn graphs. Genome Res. 18:821–829. 10.1101/gr.074492.107 18349386 PMC2336801

[8] Aziz RK Bartels D Best AA DeJongh M Disz T Edwards RA Formsma K Gerdes S Glass EM Kubal M Meyer F Olsen GJ Olson R Osterman AL Overbeek RA McNeil LK Paarmann D Paczian T Parrello B Pusch GD Reich C Stevens R Vassieva O Vonstein V Wilke A Zagnitko O 2008 The RAST server: Rapid Annotations using Subsystems Technology. BMC Genomics 9:75. 10.1186/1471-2164-9-75 18261238 PMC2265698

[9] Angiuoli SV Gussman A Klimke W Cochrane G Field D Garrity GM Kodira CD Kyrpides N Madupu R Markowitz V 2008 Toward an online repository of Standard Operating Procedures (SOPs) for (meta) genomic annotation. Omics 12:137–141. 10.1089/omi.2008.0017 18416670 PMC3196215

